# Supplementary material for: Synergism between calcium nitrate applications and fungal endophytes to increase sugar concentration in Festuca sinensis under cold stress
Source: PeerJ. 2021 Jan 7;9:e10568. doi: 10.7717/peerj.10568 (PMC8759379; doi:10.7717/peerj.10568)
Supplement: Supplemental Information 7 [file peerj-09-10568-s007.docx]

Table S1 Variance analysis results of split-split plot for root metabolic activity in *F. sinensis* under cold field conditions.

| Source | df | SS | MS | F | *p* |
| --- | --- | --- | --- | --- | --- |
| Replicate | 2 | 52.152 | 26.076 |  |  |
| Ca(NO_3_)_2_ | 2 | 437.380 | 218.690 | 460.247 | 0.000 |
| Error (a) | 4 | 1.901 | 0.475 |  |  |
| E | 1 | 90.482 | 90.482 | 168.300 | 0.000 |
| Ca(NO_3_)_2_×E | 2 | 43.324 | 21.662 | 40.292 | 0.000 |
| Error (b) | 6 | 3.226 | 0.538 |  |  |
| T | 2 | 2480.857 | 1240.429 | 1047.777 | 0.000 |
| Ca(NO_3_)_2_×T | 4 | 284.213 | 71.053 | 60.018 | 0.000 |
| E×T | 2 | 40.066 | 20.033 | 16.922 | 0.000 |
| Ca(NO_3_)_2_×E×T | 4 | 68.202 | 17.050 | 14.402 | 0.000 |
| Error (c) | 24 | 28.413 | 1.184 |  |  |
| Total | 53 |  |  |  |  |

Note: E is *Epichloë* endophyte and T is treatment time.

Table S2 Variance analysis results of split-split plot for relative water content of leaf in *F. sinensis* under cold field conditions.

| Source | df | SS | MS | F | *p* |
| --- | --- | --- | --- | --- | --- |
| Replicate | 2 | 6.969 | 3.484 |  |  |
| Ca(NO_3_)_2_ | 2 | 52.9299 | 26.465 | 10.425 | 0.026 |
| Error (a) | 4 | 10.156 | 2.539 |  |  |
| E | 1 | 57.392 | 57.392 | 13.744 | 0.010 |
| Ca(NO_3_)_2_×E | 2 | 46.479 | 23.240 | 5.565 | 0.043 |
| Error (b) | 6 | 25.054 | 4.176 |  |  |
| T | 2 | 1124.050 | 562.025 | 373.569 | 0.000 |
| Ca(NO_3_)_2_×T | 4 | 33.528 | 8.382 | 5.572 | 0.003 |
| E×T | 2 | 177.006 | 88.503 | 58.827 | 0.000 |
| Ca(NO_3_)_2_×E×T | 4 | 23.746 | 5.937 | 3.946 | 0.013 |
| Error (c) | 24 | 36.107 | 1.504 |  |  |
| Total | 53 |  |  |  |  |

Note: E is *Epichloë* endophyte and T is treatment time.

Table S3 Variance analysis results of split-split plot for total chlorophyll in *F. sinensis* under cold field conditions.

| Source | df | SS | MS | F | *p* |
| --- | --- | --- | --- | --- | --- |
| Replicate | 2 | 0.257 | 0.129 |  |  |
| Ca(NO_3_)_2_ | 2 | 0.868 | 0.434 | 254.385 | 0.000 |
| Error (a) | 4 | 0.007 | 0.002 |  |  |
| E | 1 | 0.0003 | 0.0003 | 0.046 | 0.838 |
| Ca(NO_3_)_2_×E | 2 | 0.137 | 0.068 | 11.682 | 0.009 |
| Error (b) | 6 | 0.035 | 0.006 |  |  |
| T | 2 | 5.218 | 2.609 | 857.792 | 0.000 |
| Ca(NO_3_)_2_×T | 4 | 1.461 | 0.365 | 120.014 | 0.000 |
| E×T | 2 | 0.194 | 0.097 | 31.830 | 0.000 |
| Ca(NO_3_)_2_×E×T | 4 | 0.461 | 0.115 | 37.855 | 0.000 |
| Error (c) | 24 | 0.073 | 0.003 |  |  |
| Total | 53 |  |  |  |  |

Note: E is *Epichloë* endophyte and T is treatment time.

Table S4 Variance analysis results of split-split plot for chlorophyll a/b ratio in *F. sinensis* under cold field conditions.

| Source | df | SS | MS | F | *p* |
| --- | --- | --- | --- | --- | --- |
| Replicate | 2 | 1.043 | 0.521 |  |  |
| Ca(NO_3_)_2_ | 2 | 0.262 | 0.131 | 12.557 | 0.019 |
| Error (a) | 4 | 0.042 | 0.010 |  |  |
| E | 1 | 0.024 | 0.024 | 2.779 | 0.147 |
| Ca(NO_3_)_2_×E | 2 | 0.036 | 0.018 | 2.099 | 0.204 |
| Error (b) | 6 | 0.052 | 0.009 |  |  |
| T | 2 | 5.547 | 2.774 | 141.541 | 0.000 |
| Ca(NO_3_)_2_×T | 4 | 0.129 | 0.032 | 1.651 | 0.194 |
| E×T | 2 | 0.184 | 0.092 | 4.687 | 0.019 |
| Ca(NO_3_)_2_×E×T | 4 | 0.123 | 0.031 | 1.574 | 0.214 |
| Error (c) | 24 | 0.470 | 0.020 |  |  |
| Total | 53 |  |  |  |  |

Note: E is *Epichloë* endophyte and T is treatment time.

Table S5 Variance analysis results of split-split plot for carotenoid in *F. sinensis* under cold field conditions.

| Source | df | SS | MS | F | *p* |
| --- | --- | --- | --- | --- | --- |
| Replicate | 2 | 0.008 | 0.004 |  |  |
| Ca(NO_3_)_2_ | 2 | 0.007 | 0.003 | 18.098 | 0.010 |
| Error (a) | 4 | 0.001 | 0.0002 |  |  |
| E | 1 | 0.000 | 0.000 | 0.042 | 0.845 |
| Ca(NO_3_)_2_×E | 2 | 0.002 | 0.001 | 10.575 | 0.011 |
| Error (b) | 6 | 0.0005 | 0.00008 |  |  |
| T | 2 | 0.010 | 0.005 | 32.000 | 0.000 |
| Ca(NO_3_)_2_×T | 4 | 0.018 | 0.005 | 29.495 | 0.000 |
| E×T | 2 | 0.005 | 0.002 | 15.141 | 0.000 |
| Ca(NO_3_)_2_×E×T | 4 | 0.006 | 0.001 | 9.478 | 0.000 |
| Error (c) | 24 | 0.004 | 0.0002 |  |  |
| Total | 53 |  |  |  |  |

Note: E is *Epichloë* endophyte and T is treatment time.

Table S6 Variance analysis results of split-split plot for soluble sugar of shoot in *F. sinensis* under cold field conditions.

| Source | df | SS | MS | F | *p* |
| --- | --- | --- | --- | --- | --- |
| Replicate | 2 | 0.628 | 0.314 |  |  |
| Ca(NO_3_)_2_ | 2 | 0.561 | 0.280 | 2.418 | 0.205 |
| Error (a) | 4 | 0.464 | 0.116 |  |  |
| E | 1 | 17.340 | 17.340 | 87.410 | 0.000 |
| Ca(NO_3_)_2_×E | 2 | 3.990 | 1.995 | 10.056 | 0.012 |
| Error (b) | 6 | 1.190 | 0.198 |  |  |
| T | 2 | 218.937 | 109.468 | 563.155 | 0.000 |
| Ca(NO_3_)_2_×T | 4 | 3.790 | 0.948 | 4.875 | 0.005 |
| E×T | 2 | 18.434 | 9.217 | 47.415 | 0.000 |
| Ca(NO_3_)_2_×E×T | 4 | 12.812 | 3.203 | 16.478 | 0.000 |
| Error (c) | 24 | 4.665 | 0.194 |  |  |
| Total | 53 |  |  |  |  |

Note: E is *Epichloë* endophyte and T is treatment time.

Table S7 Variance analysis results of split-split plot for soluble sugar of root in *F. sinensis* under cold field conditions.

| Source | df | SS | MS | F | *p* |
| --- | --- | --- | --- | --- | --- |
| Replicate | 2 | 0.031 | 0.016 |  |  |
| Ca(NO_3_)_2_ | 2 | 1.059 | 0.529 | 1.801 | 0.277 |
| Error (a) | 4 | 1.175 | 0.294 |  |  |
| E | 1 | 0.463 | 0.463 | 2.740 | 0.149 |
| Ca(NO_3_)_2_×E | 2 | 0.781 | 0.390 | 2.311 | 0.180 |
| Error (b) | 6 | 1.014 | 0.169 |  |  |
| T | 2 | 338.168 | 169.084 | 995.675 | 0.000 |
| Ca(NO_3_)_2_×T | 4 | 1.055 | 0.264 | 1.553 | 0.219 |
| E×T | 2 | 3.165 | 1.583 | 9.319 | 0.001 |
| Ca(NO_3_)_2_×E×T | 4 | 0.782 | 0.196 | 1.151 | 0.357 |
| Error (c) | 24 | 4.076 | 0.170 |  |  |
| Total | 53 |  |  |  |  |

Note: E is *Epichloë* endophyte and T is treatment time.

Table S8 Variance analysis results of split-split plot for sucrose of shoot in *F. sinensis* under cold field conditions.

| Source | df | SS | MS | F | *p* |
| --- | --- | --- | --- | --- | --- |
| Replicate | 2 | 0.026 | 0.013 |  |  |
| Ca(NO_3_)_2_ | 2 | 0.033 | 0.016 | 0.656 | 0.567 |
| Error (a) | 4 | 0.100 | 0.025 |  |  |
| E | 1 | 0.0007 | 0.0007 | 0.062 | 0.812 |
| Ca(NO_3_)_2_×E | 2 | 0.279 | 0.140 | 11.669 | 0.009 |
| Error (b) | 6 | 0.0718 | 0.012 |  |  |
| T | 2 | 1.286 | 0.6428 | 33.611 | 0.000 |
| Ca(NO_3_)_2_×T | 4 | 0.027 | 0.007 | 0.356 | 0.837 |
| E×T | 2 | 0.0189 | 0.009 | 0.494 | 0.616 |
| Ca(NO_3_)_2_×E×T | 4 | 0.280 | 0.070 | 3.654 | 0.018 |
| Error (c) | 24 | 0.459 | 0.019 |  |  |
| Total | 53 |  |  |  |  |

Note: E is *Epichloë* endophyte and T is treatment time.

Table S9 Variance analysis results of split-split plot for sucrose of root in *F. sinensis* under cold field conditions.

| Source | df | SS | MS | F | *p* |
| --- | --- | --- | --- | --- | --- |
| Replicate | 2 | 0.0013 | 0.0007 |  |  |
| Ca(NO_3_)_2_ | 2 | 0.069 | 0.035 | 3.150 | 0.151 |
| Error (a) | 4 | 0.044 | 0.011 |  |  |
| E | 1 | 0.006 | 0.006 | 0.904 | 0.378 |
| Ca(NO_3_)_2_×E | 2 | 0.108 | 0.054 | 8.116 | 0.020 |
| Error (b) | 6 | 0.034 | 0.007 |  |  |
| T | 2 | 27.697 | 13.848 | 1716.937 | 0.000 |
| Ca(NO_3_)_2_×T | 4 | 0.113 | 0.028 | 3.489 | 0.022 |
| E×T | 2 | 0.040 | 0.020 | 2.482 | 0.105 |
| Ca(NO_3_)_2_×E×T | 4 | 0.162 | 0.041 | 5.023 | 0.004 |
| Error (c) | 24 | 0.194 | 0.008 |  |  |
| Total | 53 |  |  |  |  |

Note: E is *Epichloë* endophyte and T is treatment time.

Table S10 Variance analysis results of split-split plot for fructose of shoot in *F. sinensis* under cold field conditions.

| Source | df | SS | MS | F | *p* |
| --- | --- | --- | --- | --- | --- |
| Replicate | 2 | 1.401 | 0.701 |  |  |
| Ca(NO_3_)_2_ | 2 | 4.862 | 2.431 | 2.621 | 0.187 |
| Error (a) | 4 | 3.709 | 0.927 |  |  |
| E | 1 | 0.839 | 0.839 | 0.724 | 0.428 |
| Ca(NO_3_)_2_×E | 2 | 3.220 | 1.610 | 1.389 | 0.319 |
| Error (b) | 6 | 6.952 | 1.1587 |  |  |
| T | 2 | 124.269 | 62.134 | 68.166 | 0.000 |
| Ca(NO_3_)_2_×T | 4 | 7.637 | 1.909 | 2.094 | 0.113 |
| E×T | 2 | 0.882 | 0.441 | 0.484 | 0.622 |
| Ca(NO_3_)_2_×E×T | 4 | 6.638 | 1.659 | 1.821 | 0.158 |
| Error (c) | 24 | 21.876 | 0.912 |  |  |
| Total | 53 |  |  |  |  |

Note: E is *Epichloë* endophyte and T is treatment time.

Table S11 Variance analysis results of split-split plot for fructose of root in *F. sinensis* under cold field conditions.

| Source | df | SS | MS | F | *p* |
| --- | --- | --- | --- | --- | --- |
| Replicate | 2 | 2.588 | 1.294 |  |  |
| Ca(NO_3_)_2_ | 2 | 0.558 | 0.279 | 0.632 | 0.577 |
| Error (a) | 4 | 1.764 | 0.441 |  |  |
| E | 1 | 2.671 | 2.671 | 7.624 | 0.033 |
| Ca(NO_3_)_2_×E | 2 | 6.318 | 3.159 | 9.016 | 0.016 |
| Error (b) | 6 | 2.102 | 0.350 |  |  |
| T | 2 | 34.945 | 17.475 | 47.395 | 0.000 |
| Ca(NO_3_)_2_×T | 4 | 5.236 | 1.309 | 3.550 | 0.021 |
| E×T | 2 | 7.957 | 3.978 | 10.790 | 0.0005 |
| Ca(NO_3_)_2_×E×T | 4 | 6.608 | 1.652 | 4.481 | 0.008 |
| Error (c) | 24 | 8.849 | 0.369 |  |  |
| Total | 53 |  |  |  |  |

Note: E is *Epichloë* endophyte and T is treatment time.

Table S12 Variance analysis results of split-split plot for glucose of shoot in *F. sinensis* under cold field conditions.

| Source | df | SS | MS | F | *p* |
| --- | --- | --- | --- | --- | --- |
| Replicate | 2 | 0.182 | 0.091 |  |  |
| Ca(NO_3_)_2_ | 2 | 0.036 | 0.018 | 2.096 | 0.238 |
| Error (a) | 4 | 0.034 | 0.009 |  |  |
| E | 1 | 0.010 | 0.010 | 1.369 | 0.286 |
| Ca(NO_3_)_2_×E | 2 | 0.073 | 0.036 | 4.902 | 0.055 |
| Error (b) | 6 | 0.045 | 0.007 |  |  |
| T | 2 | 3.264 | 1.632 | 59.602 | 0.000 |
| Ca(NO_3_)_2_×T | 4 | 0.182 | 0.045 | 1.659 | 0.192 |
| E×T | 2 | 0.096 | 0.048 | 1.745 | 0.195 |
| Ca(NO_3_)_2_×E×T | 4 | 0.502 | 0.125 | 4.582 | 0.007 |
| Error (c) | 24 | 0.657 | 0.027 |  |  |
| Total | 53 |  |  |  |  |

Note: E is *Epichloë* endophyte and T is treatment time.

Table S13 Variance analysis results of split-split plot for glucose of root in *F. sinensis* under cold field conditions.

| Source | df | SS | MS | F | *p* |
| --- | --- | --- | --- | --- | --- |
| Replicate | 2 | 0.018 | 0.009 |  |  |
| Ca(NO_3_)_2_ | 2 | 0.074 | 0.037 | 2.435 | 0.203 |
| Error (a) | 4 | 0.061 | 0.015 |  |  |
| E | 1 | 0.0002 | 0.0002 | 0.008 | 0.934 |
| Ca(NO_3_)_2_×E | 2 | 0.004 | 0.002 | 0.102 | 0.905 |
| Error (b) | 6 | 0.119 | 0.0199 |  |  |
| T | 2 | 2.750 | 1.375 | 104.878 | 0.000 |
| Ca(NO_3_)_2_×T | 4 | 0.138 | 0.034 | 2.631 | 0.059 |
| E×T | 2 | 0.0001 | 0.00007 | 0.006 | 0.995 |
| Ca(NO_3_)_2_×E×T | 4 | 0.005 | 0.001 | 0.097 | 0.982 |
| Error (c) | 24 | 0.315 | 0.013 |  |  |
| Total | 53 |  |  |  |  |

Note: E is *Epichloë* endophyte and T is treatment time.
