# Supplementary material for: Synergism between calcium nitrate applications and fungal endophytes to increase sugar concentration in Festuca sinensis under cold stress
Source: PeerJ. 2021 Jan 7;9:e10568. doi: 10.7717/peerj.10568 (PMC8759379; doi:10.7717/peerj.10568)
Supplement: Supplemental Information 8 [file peerj-09-10568-s008.docx]

Table S14 Levene’s test of equality of error variances under the same calcium nitrate treatment.

| Calcium nitrate (mM) | Dependent variable | df1 | df2 | *p* |
| --- | --- | --- | --- | --- |
| 0 | Root activity | 5 | 12 | 0.120 |
| 25 | Root activity | 5 | 12 | 0.050 |
| 50 | Root activity | 5 | 12 | 0.227 |
| 0 | Relative water content of leaf | 5 | 12 | 0.062 |
| 25 | Relative water content of leaf | 5 | 12 | 0.070 |
| 50 | Relative water content of leaf | 5 | 12 | 0.113 |
| 0 | Total chlorophyll | 5 | 12 | 0.053 |
| 25 | Total chlorophyll | 5 | 12 | 0.106 |
| 50 | Total chlorophyll | 5 | 12 | 0.273 |
| 0 | Chlorophyll a/b ratio | 5 | 12 | 0.370 |
| 25 | Chlorophyll a/b ratio | 5 | 12 | 0.051 |
| 50 | Chlorophyll a/b ratio | 5 | 12 | 0.055 |
| 0 | Carotenoid | 5 | 12 | 0.826 |
| 25 | Carotenoid | 5 | 12 | 0.452 |
| 50 | Carotenoid | 5 | 12 | 0.067 |
| 0 | Soluble sugar concentration of shoot | 5 | 12 | 0.158 |
| 25 | Soluble sugar concentration of shoot | 5 | 12 | 0.179 |
| 50 | Soluble sugar concentration of shoot | 5 | 12 | 0.518 |
| 0 | Soluble sugar concentration of root | 5 | 12 | 0.062 |
| 25 | Soluble sugar concentration of root | 5 | 12 | 0.097 |
| 50 | Soluble sugar concentration of root | 5 | 12 | 0.484 |
| 0 | Sucrose of shoot | 5 | 12 | 0.180 |
| 25 | Sucrose of shoot | 5 | 12 | 0.063 |
| 50 | Sucrose of shoot | 5 | 12 | 0.054 |
| 0 | Sucrose of root | 5 | 12 | 0.220 |
| 25 | Sucrose of root | 5 | 12 | 0.296 |
| 50 | Sucrose of root | 5 | 12 | 0.331 |
| 0 | Fructose of shoot | 5 | 12 | 0.050 |
| 25 | Fructose of shoot | 5 | 12 | 0.873 |
| 50 | Fructose of shoot | 5 | 12 | 0.078 |
| 0 | Fructose of root | 5 | 12 | 0.057 |
| 25 | Fructose of root | 5 | 12 | 0.064 |
| 50 | Fructose of root | 5 | 12 | 0.184 |
| 0 | Glucose of shoot | 5 | 12 | 0.146 |
| 25 | Glucose of shoot | 5 | 12 | 0.173 |
| 50 | Glucose of shoot | 5 | 12 | 0.245 |
| 0 | Glucose of root | 5 | 12 | 0.050 |
| 25 | Glucose of root | 5 | 12 | 0.184 |
| 50 | Glucose of root | 5 | 12 | 0.118 |

Table S15 Levene’s test of equality of error variances among a given treatment time.

| Treatment time (d) | Dependent variable | df1 | df2 | *p* |
| --- | --- | --- | --- | --- |
| 0 | Root activity | 5 | 12 | 0.761 |
| 14 | Root activity | 5 | 12 | 0.295 |
| 28 | Root activity | 5 | 12 | 0.669 |
| 0 | Relative water content of leaf | 5 | 12 | 0.007 |
| 14 | Relative water content of leaf | 5 | 12 | 0.213 |
| 28 | Relative water content of leaf | 5 | 12 | 0.724 |
| 0 | Total chlorophyll | 5 | 12 | 0.236 |
| 14 | Total chlorophyll | 5 | 12 | 0.520 |
| 28 | Total chlorophyll | 5 | 12 | 0.083 |
| 0 | Chlorophyll a/b ratio | 5 | 12 | 0.088 |
| 14 | Chlorophyll a/b ratio | 5 | 12 | 0.374 |
| 28 | Chlorophyll a/b ratio | 5 | 12 | 0.226 |
| 0 | Carotenoid | 5 | 12 | 0.496 |
| 14 | Carotenoid | 5 | 12 | 0.187 |
| 28 | Carotenoid | 5 | 12 | 0.124 |
| 0 | Soluble sugar concentration of shoot | 5 | 12 | 0.863 |
| 14 | Soluble sugar concentration of shoot | 5 | 12 | 0.078 |
| 28 | Soluble sugar concentration of shoot | 5 | 12 | 0.149 |
| 0 | Soluble sugar concentration of root | 5 | 12 | 0.077 |
| 14 | Soluble sugar concentration of root | 5 | 12 | 0.050 |
| 28 | Soluble sugar concentration of root | 5 | 12 | 0.453 |
| 0 | Sucrose of shoot | 5 | 12 | 0.704 |
| 14 | Sucrose of shoot | 5 | 12 | 0.108 |
| 28 | Sucrose of shoot | 5 | 12 | 0.180 |
| 0 | Sucrose of root | 5 | 12 | 0.170 |
| 14 | Sucrose of root | 5 | 12 | 0.584 |
| 28 | Sucrose of root | 5 | 12 | 0.249 |
| 0 | Fructose of shoot | 5 | 12 | 0.122 |
| 14 | Fructose of shoot | 5 | 12 | 0.967 |
| 28 | Fructose of shoot | 5 | 12 | 0.585 |
| 0 | Fructose of root | 5 | 12 | 0.644 |
| 14 | Fructose of root | 5 | 12 | 0.124 |
| 28 | Fructose of root | 5 | 12 | 0.374 |
| 0 | Glucose of shoot | 5 | 12 | 0.389 |
| 14 | Glucose of shoot | 5 | 12 | 0.086 |
| 28 | Glucose of shoot | 5 | 12 | 0.082 |
| 0 | Glucose of root | 5 | 12 | 0.144 |
| 14 | Glucose of root | 5 | 12 | 0.783 |
| 28 | Glucose of root | 5 | 12 | 0.242 |
