# Supplementary material for: Synergism between calcium nitrate applications and fungal endophytes to increase sugar concentration in Festuca sinensis under cold stress
Source: PeerJ. 2021 Jan 7;9:e10568. doi: 10.7717/peerj.10568 (PMC8759379; doi:10.7717/peerj.10568)
Supplement: Supplemental Information 9 — A diagram of treatment arrangement for a split-split-plot design, where three Ca(NO3)2 concentrations (0, 25 and 50 mmol/L, red colour) were randomly allocated in three replicates as main plot treatments and two endophyte types (E+ and E–, with and without endophyte infection) were randomly allocated in the main plots as split plot treatments. In each of the three sampling times (0, 14 and 28 d, blue colour), two plants were randomly sampled from a split-split plot (i.e., combinations of endophyte and Ca(NO3)2 concentration). [file peerj-09-10568-s009.docx]

|  | Replicate I | | | |  | Replicate II | | | |  | Replicate III | | | |  |
| --- | --- | --- | --- | --- | --- | --- | --- | --- | --- | --- | --- | --- | --- | --- | --- |
| Split plot I | E+0 | E–0–0 | E+0+28 | E–0 |  | E–25 | E+25+28 | E–25–14 | E+25 |  | E+50 | E–50–14 | E+50+0 | E–50 | |
|  | E+0 | E–0–14 | E+0+0 | E–0 |  | E–25 | E+25+0 | E–25–28 | E+25 |  | E+50 | E–50–28 | E+50+14 | E–50 | |
|  | E+0 | E–0–28 | E+0+14 | E–0 |  | E–25 | E+25+14 | E–25–0 | E+25 |  | E+50 | E–50–0 | E+50+28 | E–50 | |
|  | E+0 | E–0–0 | E+0+28 | E–0 |  | E–25 | E+25+28 | E–25–14 | E+25 |  | E+50 | E–50–14 | E+50+0 | E–50 | |
|  | E+0 | E–0–14 | E+0+0 | E–0 |  | E–25 | E+25+0 | E–25–28 | E+25 |  | E+50 | E–50–28 | E+50+14 | E–50 | |
|  | E+0 | E–0–28 | E+0+14 | E–0 |  | E–25 | E+25+14 | E–25–0 | E+25 |  | E+50 | E–50–0 | E+50+28 | E–50 | |
|  |  |  |  |  |  |  |  |  |  |  |  |  |  |  | |
| Split plot II | E–25 | E+25+14 | E–25–0 | E+25 |  | E+50 | E–50–0 | E+50+28 | E–50 |  | E–0 | E+0+28 | E–0–14 | E+0 | |
|  | E–25 | E+25+28 | E–25–14 | E+25 |  | E+50 | E–50–14 | E+50+0 | E–50 |  | E–0 | E+0+0 | E–0–28 | E+0 | |
|  | E–25 | E+25+0 | E–25–28 | E+25 |  | E+50 | E–50–28 | E+50+14 | E–50 |  | E–0 | E+0+14 | E–0–0 | E+0 | |
|  | E–25 | E+25+14 | E–25–0 | E+25 |  | E+50 | E–50–0 | E+50+28 | E–50 |  | E–0 | E+0+28 | E–0–14 | E+0 | |
|  | E–25 | E+25+28 | E–25–14 | E+25 |  | E+50 | E–50–14 | E+50+0 | E–50 |  | E–0 | E+0+0 | E–0–28 | E+0 | |
|  | E–25 | E+25+14 | E–25–28 | E+25 |  | E+50 | E–50–28 | E+50+14 | E–50 |  | E–0 | E+0+14 | E–0–0 | E+0 | |
|  |  |  |  |  |  |  |  |  |  |  |  |  |  |  | |
| Split plot III | E+50 | E–50–28 | E+50+14 | E–50 |  | E–0 | E+0+14 | E–0–0 | E+0 |  | E+25 | E–25–0 | E+25+28 | E–25 | |
|  | E+50 | E–50–0 | E+50+28 | E–50 |  | E–0 | E+0+28 | E–0–14 | E+0 |  | E+25 | E–25–14 | E+25+0 | E–25 | |
|  | E+50 | E–50–14 | E+50+0 | E–50 |  | E–0 | E+0+0 | E–0–28 | E+0 |  | E+25 | E–25–28 | E+25+14 | E–25 | |
|  | E+50 | E–50–28 | E+50+14 | E–50 |  | E–0 | E+0+14 | E–0–0 | E+0 |  | E+25 | E–25–0 | E+25+28 | E–25 | |
|  | E+50 | E–50–0 | E+50+28 | E–50 |  | E–0 | E+0+28 | E–0–14 | E+0 |  | E+25 | E–25–14 | E+25+0 | E–25 | |
|  | E+50 | E–50–14 | E+50+0 | E–50 |  | E–0 | E+0+0 | E–0–28 | E+0 |  | E+25 | E–25–28 | E+25+14 | E–25 | |

**Figure S1** A diagram of treatment arrangement for a split-split-plot design, where three Ca(NO_3_)_2_ concentrations (0, 25 and 50 mmol/L, red colour) were randomly allocated in three replicates as main plot treatments and two endophyte types (E+ and E–, with and without endophyte infection) were randomly allocated in the main plots as split plot treatments. In each of the three sampling times (0, 14 and 28 d, blue colour), two plants were randomly sampled from a split-split plot (i.e., combinations of endophyte and Ca(NO_3_)_2_ concentration).
